# Evaluation of association of common variants in *HTR1A* and *HTR5A* with schizophrenia and executive function

**DOI:** 10.1038/srep38048

**Published:** 2016-11-29

**Authors:** Fanglin Guan, Huali Lin, Gang Chen, Lu Li, Teng Chen, Xinshe Liu, Jiuqiang Han, Tao Li

**Affiliations:** 1 Department of Forensic Psychiatry, School of Medicine & Forensics, Xi’an Jiaotong University, Xi’an, China; 2 Key Laboratory of National Ministry of Health for Forensic Sciences, School of Medicine & Forensics, Xi’an Jiaotong University, Xi’an, China; 3 Xi’an Mental Health Center, Xi’an, China; 4 Department of Forensic Medicine, School of Medicine & Forensics, Xi’an Jiaotong University, Xi’an, China; 5 School of Electronic and Information Engineering, Xi’an Jiaotong University, Xi’an, China

## Abstract

The 5-HT1A receptor (HTR1A) and the 5-HT5A receptor (HTR5A) are key 5-HT receptors with distinct inhibitory functions. Studies have been conducted to investigate the association of a few *HTR1A* polymorphisms with schizophrenia, producing conflicting results, and the relationship between *HTR5A* and schizophrenia has not yet been well investigated. We aimed to examine the association of *HTR1A* and *HTR5A* with schizophrenia and executive function. The study included a discovery stage with 1,115 patients and 2,289 controls and a replication stage with 2,128 patients and 3,865 controls. A total of 30 common SNPs in *HTR1A* and *HTR5A* were genotyped in the discovery stage, and significantly associated SNPs were genotyped in the replication stage. We identified that two SNPs (rs878567 in *HTR1A* and rs1800883 in *HTR5A*) were significantly associated with schizophrenia in both datasets, and similar results were observed in imputation and haplotype association analyses. Moreover, we found that SNP rs1800883 significantly interacted with executive function when processing the perseverative error of Wisconsin Card Sorting Test in patients. Our results provide further supportive evidence of the effect of *HTR1A* and *HTR5A* on the etiology of schizophrenia and suggest that the selected genetic variations in *HTR5A* may be involved in impaired executive function.

Schizophrenia (SCZ) is a complex severe chronic psychiatric disorder with a heterogeneous clinical phenotype, and it is generally thought to have a worldwide uniform lifetime morbid risk of 1% across time, geography and gender^1^. Many epidemiological studies and recent genetic studies of association mapping have suggested genetic and gene-environment interactions account for over 80% of the susceptibility in the development of SCZ^2^,^3^,^4^, and these studies have been accelerated by the widely used application of genome-wide association studies (GWAS) on SCZ^5^,^6^,^7^,^8^. A recent SCZ study by PGC reported that 108 loci met genome-wide significance from a multi-stage GWAS with 36,989 cases and 113,075 controls. Among those loci, 83 of them were reported for the first time^9^. However, current replicable GWAS results account for only a small percentage of the estimated heritability^10^, and their systematic biological interpretation is lacking. The results of GWAS contain many false positives^11^, and genetic heterogeneity of SCZ exists in different populations^12^. Additionally, false negative results commonly exist in GWAS as well, because from recent GWAS data it has been seen that the enlargement of the sample size is intimately related to the power to detect variants of small effect^13^,^14^. Thus, although GWAS provide a promising approach for studying the genetics of SCZ, follow-up studies are necessary to confirm GWAS findings and extend them.

In the present etiological hypothesis for SCZ, the mainstream has been neurotransmitter abnormality, which includes dopamine^15^, glutamic acid^16^, γ-aminobutyric acid^17^, and serotonin (5-HT)^18^. Among them, 5-HT acts as a modulator in the dopamine release and a regulator of brain development^19^, and 5-HT signaling in the brain has been considered an important component of the pathogenesis of SCZ^18^. Consequently, 5-HT receptor genes have been suggested to be associated with genetic susceptibility to SCZ and cognitive function^20^,^21^,^22^. Additionally, despite the possible complex interactions of 5-HT signaling with dopamine and other neurotransmitter systems in the brain, genetic variants in 5-HT receptors have been found to play important roles in the development and treatment of SCZ^23^,^24^,^25^. As is known, there are seven families of 5-HT receptors. Among them, both the 5-HT1A receptor (HTR1A) and the 5-HT5A receptor (HTR5A) are key serotonin receptors with distinct inhibitory functions. HTR1A is the most abundantly expressed 5-HT receptor subtype in the mammalian brain, and HTR5A is expressed in the central nervous system, a significant portion of cortical pyramidal neurons^26^,^27^. Previous studies have investigated the association between only a few *HTR1A* variants and SCZ, but they have produced conflicting results^28^,^29^,^30^,^31^,^32^,^33^,^34^,^35^. So far, *HTR5A* has been studied less than other *HTRs*. Several studies have implicated *HTR5A* in the control of circadian rhythms, mood and cognitive function and in genetic associations with bipolar disorder and major depression^36^,^37^. The relationship between *HTR5A* and SCZ is still understood poorly. Given that the additive effect of many minor genes contributes to the disease occurrence and unknown biological mechanisms of *HTR1A* and *HTR5A* involved in the pathophysiology of SCZ, it remains necessary to systematically explore the potential association of *HTR1A* and *HTR5A* with SCZ in independent populations with larger samples.

In addition, 5-HT dysfunction has been demonstrated to contribute to not only emotional difficulty but also cognition deficit^38^. The association between the 5-HT system and cognitive dysfunction has been investigated in SCZ patients in some recent studies^39^,^40^,^41^, but *HTR1A* and *HTR5A* have not yet been evaluated. Given of the heterogeneous nature of SCZ and inconsistent findings from genetic association studies of SCZ, the use of endophenotypes to refine the SCZ phenotype has been advocated^42^. Executive functioning, as measured by the Wisconsin Card Sorting Test (WCST), is known to be impaired in patients with SCZ^43^, and substantial empirical evidence shows executive dysfunction is a potential endophenotypic marker. In addition, perseverative deficits in executive function have been considered not only to associate with frontal dysfunction in SCZ^44^ but also to serve as a marker of vulnerability to SCZ^45^. Thus, the index of perseverative errors in WCST has been widely used as SCZ endophenotypes and as a composite profile of neurocognitive performance^46^, which would be helpful in addressing the heterogeneity of the disorder in genetic studies.

This study was first designed to assess evidence of the association with SCZ of common variants in *HTR1A* and *HTR5A* using two-stage independent samples from the Han Chinese population. Furthermore, to better understand the relationship of *HTR1A* and *HTR5A* with executive function under pathological and non-pathological conditions, our secondary purpose was to evaluate the relationship of the schizophrenia-associated variants in *HTR1A* and *HTR5A* with four WCST indices (perseverative errors, non-perseverative errors, total errors and trials to complete the first category) in SCZ patients and healthy individuals.

## Materials and Methods

### Subjects

Two independent datasets were included in our study. In the discovery stage, we recruited 1,115 patients and 2,289 unrelated healthy controls. In the replication stage, 5,993 subjects consisted of 2,128 patients and 3,865 controls. The characteristics of the study subjects are summarized in Table 1. All patients were recruited from the inpatient and outpatient clinical services of a psychiatric unit at Xi’an Mental Health Center, and the diagnosis of SCZ was confirmed by at least two experienced psychiatrists based on the Diagnostic and Statistical Manual of Mental Disorders (DSM-IV) criteria for SCZ^47^. Patients with substance-induced psychotic disorders, learning disabilities, head injuries, and other symptomatic psychoses were excluded from the present study. A total of 2,128 patients and 2,794 controls in the replication dataset completed the cognitive assessments, and relevant index parameters were obtained. All patients were at clinical stability when the Positive and Negative Syndrome Scale (PANSS) and WCST were administered. For SCZ patients in the replication stage, the information on the duration of the illness and the Total PANSS scores are presented in Table 1. All healthy subjects were enrolled from the city of Xi’an in Shaanxi Province based on the selection criteria of frequency-matched age (±5 years) and gender of the patients. Trained psychiatrists interviewed the healthy controls individually using the Structured Clinical Interview for DSM-IV-TR Axis I Disorders-Nonpatient Edition (SCID-NP)^47^, and all controls self-reported as being free from physical diseases and from any individual or family history of mental illness. All subjects were of Han descent, and based on self-reports regarding their own and their paternal grandparents’ place of birth, we excluded anyone who was not born locally or whose families within the past three generations were not born locally. All participants completed written informed consent forms.

### Ethics statements

All procedures were conducted in accordance with the ethical standards of the responsible committee on human experimentation (Committee on Human Rights Related to Research Involving Human Subjects, Xi’an Jiaotong University) and the Helsinki Declaration of 1975, revised in 2008. This research was approved by the Medical Ethics Committee of Xi’an Jiaotong University.

### Executive function assessment

Executive performance was assessed using a computerized version of the WCST. WCST is a standard test used to assess executive function. It requires participants to match a target card to one of four alternatives. During the WCST, subjects were required to match 128 response cards to 4 stimulus cards in one of three dimensions (color, form, or number) by pressing one of the one to four number keys on the computer keyboard. After each match, the participants received correct or incorrect feedback on the screen and needed to understand the underlying principle governing the matching rule. After 10 consecutive correct matches, the rule shifted without any announcement, and the participants were required to adjust their strategy accordingly. Four indices in the WCST were included for subsequent analyses: (1) perseverative errors (PE): number of errors that were perseverative; (2) non-perseverative errors (NPE): number of errors that were not perseverative; (3) total errors (TE): total numbers of perseverative and non-perseverative errors; and (4) trials to complete first category (TCFC): number of trials to successfully complete the first category.

### SNPs selection and genotyping

We searched for all SNPs with minor allele frequencies (MAF) ≥ 0.01 between 5 kb upstream and 5 kb downstream (10 kb window) of *HTR1A* and *HTR5A* in the HapMap CHB database by Haploview v4.2. We found 8 SNPs (rs1423691, rs10052087, rs878567, rs6449693, rs34118353, rs6294, rs6295, rs75604552) in *HTR1A* and 22 SNPs (rs1440454, rs9642644, rs2919435, rs2581842, rs2873379, rs1017488, rs1881691, rs1800883, rs6320, rs2241859, rs2581841, rs1561598, rs732050, rs2698512, rs1657268, rs1730208, rs1946915, rs1631327, rs2581831, rs1371818, rs2581832, rs1730215) in *HTR5A.* Therefore, those 30 SNPs that completely covered the region of the *HTR1A* and *HTR5A* were included in our further analyses. Genomic DNA was extracted from peripheral blood leukocytes according to the manufacturer’s protocol (Genomic DNA kit, Axygen Scientific Inc., California, USA). DNA was stored at −20 °C for SNP analyses. Genotyping was performed for all SNPs using the MassARRAY platform (Sequenom, San Diego, California, US). Briefly, SNPs were genotyped using high-throughput, matrix-assisted laser desorption ionization–time-of-flight (MALDI–TOF) mass spectrometry. Next, the resulting spectra were processed using Typer Analyzer software (Sequenom, San Diego, California, US), and genotype data were generated from the samples. The final genotype call rate of each SNP was greater than 99.4%, and the overall genotyping call rate was 99.8%. Additionally, 5% of random samples were repeated, and the results were 100% concordant. Thus, the reliability of further statistical analyses was ensured.

### Statistical analyses

We used Haploview v4.2 to conduct the Hardy-Weinberg equilibrium (HWE) test for each SNP in samples and to calculate the minor allele frequency (MAF) for each marker in both the discovery and replication stages. We implemented a logistic model using the genetic analysis software Plink v1.9 to investigate the association between genetic polymorphisms and disorder status in both stages, and the age and gender of subjects were included in the model as two covariates to account for potential confounding effects. The Bonferroni correction was applied to address the multiple comparison problems (corrected *P* = *P* × N). To increase the density of the SNP markers in the discovery dataset, we implemented imputation using genetic software IMPUT2 with the HapMap dataset from combined sample set (CHB and JPT) as the reference. The follow-up association analyses were performed with software SNPTEST v2. We utilized the parameter of “average certainty” calculated in IMPUTE2 as the main indicator of imputation quality. The threshold of this indicator was chosen by exploring the patterns of Q-Q plots based on the *P*-values of the results of the association analyses based on multiple marker sets obtained using different certainty thresholds. Haploview v4.2 software was also utilized to investigate the linkage disequilibrium (LD) structure of the candidate markers. LD blocks were constructed using the default algorithm taken from Gabriel *et al*.^48^. We generated 95% confidence bounds on *D*′, and each comparison was called “strong LD” when the confidence bounds had an upper bound ≥0.98 and a lower bound ≥0.7. A block was created if 95% of the informative comparisons were “strong LD”. Haplotype frequency was calculated using GENECOUNTING v2.2 software. All common haplotypes (frequency > 0.01) were selected for significant test through a likelihood ratio test, followed by permutation testing. The global *P*-values of haplotype analyses were calculated based on the differences in the haplotypic frequencies distribution among patients and controls. Multivariate analyses were performed to investigate the genotype-phenotype correlation of schizophrenia-associated variants in *HTR1A* and *HTR5A* with WCST parameters of patients and controls in the replication stage considering age, gender, duration of illness and PANSS scores as covariates. All tests performed were two-tailed, and a *P*-value of 0.05 was chosen as the threshold for significance in all analyses. We used PGA v2.0 to perform the power calculations. In our study, depending on the minor allele frequencies, the statistical power to detect a risk allele with an effect size of 1.5 ranged from 0.75 to 0.90 and 0.85 to 0.94 at a false positive rate of 5% in the discovery and replication stage, respectively.

## Results

### Allelic and genotypic association analyses

**Figure 1:**
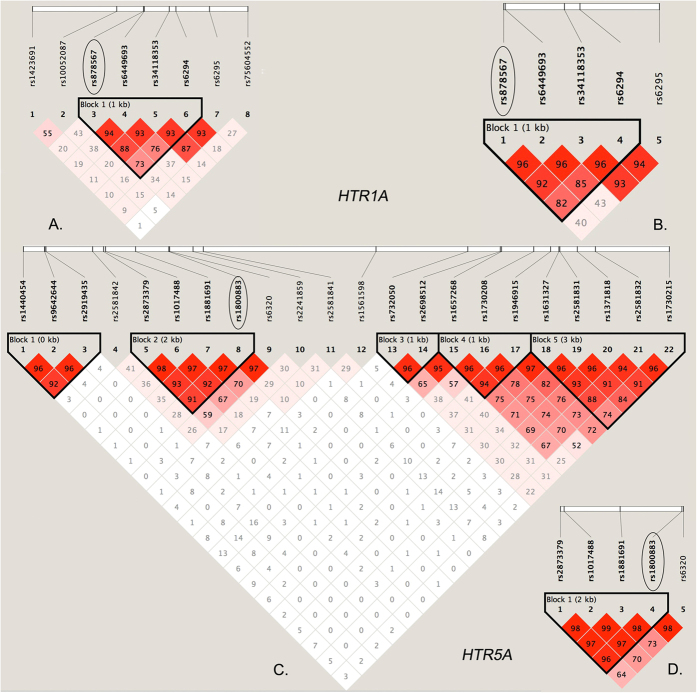
LD structure and significantly associated LD blocks based on two-stage data. LD blocks in *HTR1A* are indicated by the shaded matrices, and significantly associated LD block 1 in the discovery stage (**A**) was confirmed in the replication stage (**B**); LD blocks in *HTR5A* are indicated by the shaded matrices, and significantly associated LD block 2 in the discovery stage (**C**) was also confirmed in the replication stage (**D**). The two associated SNPs (rs878567 and rs1800883) are marked by circles.

A total of 30 SNPs within *HTR1A* and *HTR5A* were genotyped in the discovery dataset. The allele and genotype frequencies of all SNPs in cases and controls, as well as the HWE test, are presented in Table 2 and Table S1. All SNPs were in HWE in cases and controls. As shown in Table 2, two SNPs (rs878567 and rs6295) in the *HTR1A* and two SNPs (rs1800883 and rs6320) in the *HTR5A* were associated with SCZ (allelic *P*-value = 0.001541, 0.018648, 0.000264 and 0.017216, respectively). However, only rs878567 in the *HTR1A* and rs1800883 in the *HTR5A* showed a significant association with SCZ after the Bonferroni correction (corrected allelic *P*-value = 0.04623 and 0.00792) (Table 2). Genotypic association analyses confirmed the results with a similar pattern (Table 2). There were no significant differences in the other 26 SNPs in the allelic or genotypic analyses (Table S1). Given of the small effect sizes conferred by common alleles, a given SNP is best replicated in independent larger samples. In the replication dataset, we performed the second-stage single SNP association analyses for the 4 SNPs (rs878567, rs6295, rs1800883 and rs6320) together with another 6 SNPs (rs6449693, rs34118353, rs6294, rs2873379, rs1017488 and rs1881691) in the two different LD blocks including rs878567 and rs1800883, respectively (Fig. 1). The significant association of rs878567 and rs1800883 with SCZ was replicated (corrected allelic *P*-value = 0.00508 and 0.01095) (Table 2). The similar results were also confirmed by genotypic association analyses. The other 8 SNPs did not still differ significantly in their allelic or genotype distributions (Table S1).

### Imputation and haplotypic association analyses

**Figure 2:**
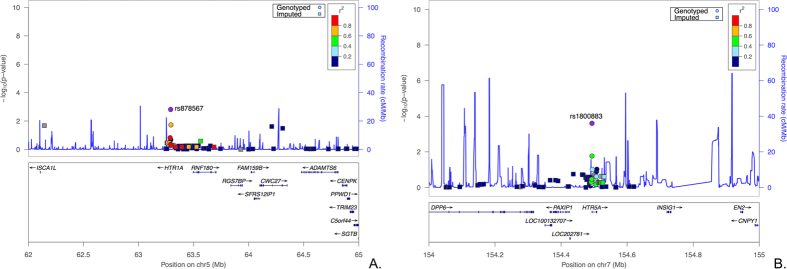
Regional association plots based on imputed region covering *HTR1A* and *HTR5A*. Imputed SNPs are indicated with a circle, and genotyped SNPs are indicated with a square. The most significant genotyped SNPs were chosen as reference SNPs in the plots (rs878567 and rs1800883).

The imputation was implemented for two 5-Mb genomic regions including *HTR1A* and *HTR5A*, respectively. We chose 0.8 as the average certainty threshold to exclude those potential imputed SNPs with low accuracy. This threshold was chosen by exploring the patterns of Q-Q plots based on the *P*-values of the association analyses based on multiple marker sets obtained using different certainty thresholds (Figure S1). We focused only on the common SNPs, so MAF ≥ 0.01 was applied as another filter criterion. After applying these filters, a total of 162 SNPs (82 SNPs in the *HTR1A* and 80 SNPs in the *HTR5A*) were successfully imputed and tested for their association based on the data of 30 genotyped SNPs in the discovery stage. We presented the imputed SNPs with significance (*P* < 0.05) from the association analyses in Table S2, and the results of the association analyses from the two imputed genomic regions indicated that the two SNPs (rs878567 and rs1800883) were still the most significant (Fig. 2).

Generally, haplotype based analysis can be more statistically powerful than single SNP based association analysis especially when the haplotypes were formed by several highly correlated SNPs^49^,^50^. Thus, we implemented haplotype based analyses in order to further validate the significant signals from single-marker analyses. To perform haplotype-based association analyses, we examined LD structure within the genotype data of 30 SNPs from the discovery stage. We identified one haplotype block in the *HTR1A* (Fig. 1A) and five blocks in the *HTR5A* (Fig. 1C). As Fig. 1 shows, two significantly associated SNPs (rs878567 and rs1800883) were located in the two different haplotype blocks. The significant *P* values (global *P*-value < 0.001) were both obtained in the two blocks through the association test of the haplotype, and some specific haplotypes in the two blocks showed risk associations with SCZ (GAGC in *HTR1A*; TCAG and TACG in *HTR5A*). The results provided further evidence of significant association of rs878567 and rs1800883 with SCZ (Table 3). These significant results were also further confirmed in the replication stage (Table 3), where the two blocks including rs878567 and rs1800883 were also constructed (Fig. 1B,D, respectively).

### Genotype-phenotype correlation of associated SNPs with executive function

To investigate the genotype-phenotype correlation of the two schizophrenia-associated SNPs (rs878567 in *HTR1A* and rs1800883 in *HTR5A*) with executive function in patients and controls, we conducted multivariate analyses of PE, NPE, TE and TCFC for each genotype group of the two associated SNPs in the replication dataset. As shown in Table 4, there were significant differences in 3 index parameters (PE, TE and TCFC) for only rs1800883 in patients after the adjustment for age, gender, duration of illness and PANSS scores, and there was better executive function performance in the C allele carriers than in the patients with the homozygote for the G risk allele. In healthy controls, we found no significant differences for the 2 SNPs in all index parameters (PE, NPE, TE and TCFC).

## Discussion

So far, some studies have investigated the association of HTR1A gene with SCZ in different populations including American, Korean, Japanese and Han Chinese populations^28^,^29^,^30^,^31^,^32^,^33^,^34^,^35^, and the significant association was found in American^29^, Japanese^33^ and Han Chinese populations^34^,^35^. Thus, the association of HTR1A gene with SCZ is not specific to a certain ethnic group. Although rs878567 was found to have no significant association with SCZ in the studies of Kishi *et al*.^33^ and Zhou *et al*.^34^, the significant signal of rs878567 was captured in the studies of Lin *et al*.^35^ and ours. It is worth noting that a trend of allelic and genotypic association signals in rs878567 was reported by Kishi *et al*. to be stronger than that in rs6295^33^ and the number of samples enrolled in the study by Zhou *et al*. was very small^34^. Kishi *et al*. also demonstrated that rs878567 was significantly associated with methamphetamine (METH)-induced psychosis, which indicated that the susceptibility of rs878567 to SCZ was more influenced by some factors than that of rs6295^32^. Although Lemonde *et al*. suggested that rs6295 might regulate the expression of the HTR1A gene to reduce serotonergic transmission^51^, the molecular mechanism of the regulation is still unknown. Some postmortem studies have demonstrated that 5-HT1A receptor was overexpressed in the prefrontal cortex of SCZ patients^52^,^53^. These results indicated that abnormalities in the 5-HT1A receptor could alter serotonergic neural transmission, which may be involved in the development of SCZ^18^. As our results show, the LD block consisted of rs878567-rs6449693-rs34118353-rs6294, covering a wide and important region, including the exon region in HTR1A. To date, there has been no known functional study on the SNP of rs878567 located in the *HTR1A* 3′ untranslated region (UTR). Thus, we could hypothesize that the SNP might influence the transcription/translation of HTR1A in a certain unpredicted way, or it may be in LD with other undiscovered variants involved with the regulation machinery conferring the risk for SCZ. Given that it is possible for rs878567 to influence the biological function/pathway of the *HTR1A* gene in the brain, we suggest that a functional analysis of rs878567 should be conducted in future studies.

Until recently, *HTR5A* was not well investigated. It has been reported that the genetic deletion of HTR5A could induce abnormal behavior in stress tests and inappropriate responses to novel situations in an HTR5A knockout mouse^54^. Another study recently indicated that *HTR5A* expression levels remained stable throughout development but were significantly increased in adulthood^55^. Furthermore, Birkett *et al*.^36^ found a strong association between *HTR5A* and major depressive disorder, and Yosifova *et al*.^37^ also reported a significant association between the *HTR5A* gene and bipolar susceptibility. The genetic risk variants were found to be substantially shared between SCZ and bipolar disorder (high genetic mean ± standard error correlation, 0.68 ± 0.04) and between SCZ and major depressive disorder (moderate genetic correlation, 0.47 ± 0.06)^56^. Our study reported the significant association of HTR5A gene with SCZ for the first time. Although the SNP of rs1800883 is located in the promoter region of HTR5A gene, it is still unknown whether the SNP is a causative variant in the etiology of SCZ and has a direct biological effect on the HTR5A or is just a surrogate of untyped variants. Another recent study found a significant association of interactions of *HTR5A* with other serotonergic genes^57^. Therefore, given the region around the SNP of rs1800883 in the *HTR5A* is enriched in CpG repeats, it could be hypothesis generating and of interest for future studies on epigenetic control by methylation, although there has been no functional study concerning the SNP to date, and a targeted sequencing-based study design may provide more insight. In the recently published large scale Psychiatric Genomics Consortium (PGC) GWAS^9^, neither *HTR1A* nor *HTR5A* were identified to be independently associated with SCZ. Given that GWAS has a larger genomic coverage, its results are susceptible to many type I errors. Additionally, the stringent *P*-value used in the GWAS to correct for multiple comparison problems will result in the loss of several signals with moderate effects. Our study design avoids this disadvantage by focusing on a couple of functionally related genes and controlling the number of genotyped markers to a reasonable level. Although our findings provide the potential functional significance of rs878567 and rs1800883 from different perspectives, our preliminary results need to be confirmed in different populations and supported by biological evidence in future research.

Recently, endophenotype approaches have been applied in SCZ research. Although it has been reported that endophenotype is heritable and segregates with known risk loci of SCZ^42^, it is not a valid affirmation for any potential endophenotype, and further investigations would be desirable for a specific endophenotype. The application of WCST performance deficits can be considered endophenotypic markers for SCZ susceptibility because the endophenotypic nature of this deficit may help index underlying genetic liability or vulnerability of the disease^58^. A previous study also provided supportive evidence that subjects rating high on the Schizotypal Personality Questionnaire (SPQ)^59^ showed more perseverative errors reflecting set-shifting problems than those scoring average on the SPQ^60^. However, in our study, there is not an increased probability of association for the associated SNPs when considering the executive function measures with respect to the SCZ phenotype. One possible explanation is the lack of adequate adjustments for education duration and medication treatment on WCST performance, which may be associated with WCST performance. Although stimulation of the 5-HT1A receptor has been known to reduce cognitive dysfunction in SCZ^61^, our results revealed no correlation between the schizophrenia-associated SNP of rs878567 in *HTR1A* and phenotypes in WCST. By contrast, the associated SNP of rs1800883 in *HTR5A* was found to have a significant association with phenotypes in WCST in patients but not in healthy controls. In contrast with the non-perseverative error, the perseverative errors outcome is a measurement of perseveration and flexibility^62^, and it is used traditionally as a measure of set-shifting abilities^63^. Our study for the first time indicated the involvement of the *HTR5A* in set-shifting abilities in SCZ patients. Working memory may contribute to set-shifting impairment in SCZ patients, as shown for SCZ at onset and during the chronic phase of the disease^64^. Therefore, because of the lack working memory measures in our study, we did not completely rule out the possibility that the association of the *HTR5A* with perseverative errors might have been mediated by working memory, which would be required for future research to clarify. Although our study supported the effect of the *HTR5A* in cognitive set-shifting abilities and the role of SNP rs1800883 as either a causative variant or a biomarker associated with others, the exact mechanisms involved remain to be investigated. The serotonergic system influences almost every sphere of mammalian physiology, including the maintenance of mood, cognition, learning, and memory^21^, and changes to the expression or function of HTRs have been shown to occur as a result of genetic polymorphisms^65^. As is known, the molecular mechanism of cognitive deficits is very complex, and many genes, pathways and signals have been confirmed to be involved in the process. Disrupting a normal balance in the effects of serotonin on the prefrontal cortex during a vulnerable developmental period may result in abnormal emotional regulation, cognition, and behavior in the short term. Due to the complex network of 5-HT signaling in the brain and interactions with dopamine and other neurotransmitter systems, investigations of potential epistatic interaction with other genes would be desirable.

Additionally, some potential limitations of our study should be kept in mind. First, although our moderate sample size was larger than most of individual association studies, given of the smaller observed effect sizes of the associated SNPs in our study, the statistical power to detect the moderate effect sizes was not strong compared with GWASs. Thus, the significant association signals captured in our study need to be confirmed in future studies with enlarged sample size. Moreover, given that SNPs and haplotype structures can vary among different ethnic groups, our significant results should also be replicated and validated in other ethnic groups. Second, we could not conclusively rule out some other potential confounders, such as education duration, medication history and drugs in treatment, which might be influenced by the risk variant in association with the influence on executive ability. Finally, although executive functioning is impaired in SCZ, there are some debates concerning the independence of executive functioning from constructs such as fluid intelligence. In the present study, measurements of fluid intelligence, intelligence quotient, n-back and other cognitive measures were not conducted. Therefore, our results regarding executive function should be considered preliminary and require further confirmation and more supportive evidence in future research.

In summary, our results provide supportive evidence of *HTR1A* and *HTR5A* for the association with SCZ. However, their mechanisms involved in the etiology of SCZ remain poorly understood. Given the molecular basis of the associations within the complex network underlying the etiology and pathophysiology of SCZ, additional follow-up studies are required that include high-density mapping and deep sequencing in other populations to confirm our findings and explain the specific mechanisms.

## Additional Information

**How to cite this article**: Guan, F. *et al*. Evaluation of association of common variants in *HTR1A* and *HTR5A* with schizophrenia and executive function. *Sci. Rep.* **6**, 38048; doi: 10.1038/srep38048 (2016).

**Publisher's note:** Springer Nature remains neutral with regard to jurisdictional claims in published maps and institutional affiliations.

## Supplementary Material

Supplementary Information

## Figures and Tables

**Table 1:** Characteristic information of study subjects.

Sample stage	Parameters	Case	Control
The Discovery Stage	Male (%)	535 (47.98)	1107 (48.36)
	Female (%)	580 (52.02)	1182 (51.64)
	Total	1115	2289
	Mean age (SD)	36.49 (6.78)	36.42 (9.23)
	Age range (years)	18–47	18–47
The Replication Stage	Male (%)	1030 (48.40)	1863 (48.20)
	Female (%)	1098 (51.60)	2002 (51.80)
	Total	2128	3865
	Mean age (SD.)	35.38 (10.39)	35.53 (11.38)
	Age range (years)	18–57	18–57
	Duration of illness (years) (SD)	5.43 (2.62)	
	Total PANSS scores (SD)	63.21 (11.19)	

SD: standard deviation; PANSS: Positive and Negative Syndrome Scale.

**Table 2:** Allele and genotype frequency of single SNP association analysis.

Dataset	Gene	SNP Affection	H-WE	OR^2^ 95% CI	Allelic count (Freq. %)		Allelic *P*-value^1^	Genotype count (Freq. %)			Genotypic *P*-value^1^
The discovery dataset	*HTR1A*	rs878567			***G***	A		GG	GA	AA	
		SCZ	0.113	1.24	1843(82.65)	387(17.35)	***0.001541***	754(67.62)	335(30.04)	26(2.33)	***0.001584***
		CTR	0.453	1.08–1.41	3635(79.4)	943(20.6)	***0.04623***	1449(63.3)	737(32.2)	103(4.5)	***0.04752***
		rs6295			C	G		CC	CG	GG	
		SCZ	0.159	1.15	1711(76.73)	519(23.27)	***0.018648***	648(58.12)	415(37.22)	52(4.66)	***0.024294***
		CTR	0.634	1.02–1.30	3392(74.09)	1186(25.91)	0.55944	1261(55.09)	870(38.01)	158(6.9)	0.72882
	*HTR5A*	rs1800883			C	***G***		CC	CG	GG	
		SCZ	0.339	1.21	1083(48.57)	1147(51.43)	***0.000264***	255(22.87)	573(51.39)	287(25.74)	***0.000899***
		CTR	0.847	1.09–1.34	2439(53.28)	2139(46.72)	**0.00792**	652(28.48)	1135(49.58)	502(21.93)	***0.02697***
		rs6320			T	A		TT	TA	AA	
		SCZ	0.755	1.14	1453(65.16)	777(34.84)	***0.017216***	471(42.24)	511(45.83)	133(11.93)	0.055259
		CTR	0.807	1.02–1.26	2847(62.19)	1731(37.81)	0.51648	888(38.79)	1071(46.79)	330(14.42)	
The replication dataset	*HTR1A*	rs878567			***G***	A		GG	GA	AA	
		SCZ	0.479	1.19	3508(82.42)	748(17.58)	***0.000508***	1441(67.72)	626(29.42)	61(2.87)	***0.002009***
		CTR	0.969	1.08–1.31	6169(79.81)	1561(20.19)	***0.00508***	2462(63.7)	1245(32.21)	158(4.09)	***0.02009***
		rs6295			C	G		CC	CG	GG	
		SCZ	0.366	1.09	3243(76.2)	1013(23.8)	0.068321	1228(57.71)	787(36.98)	113(5.31)	0.106148
		CTR	0.522	0.98–1.18	5774(74.7)	1956(25.3)		2164(55.99)	1446(37.41)	255(6.6)	
	*HTR5A*	rs1800883			C	***G***		CC	CG	GG	
		SCZ	0.463	1.13	2111(49.6)	2145(50.4)	***0.001095***	532(25)	1047(49.2)	549(25.8)	***0.003746***
		CTR	0.841	1.05–1.22	4075(52.72)	3655(47.28)	***0.01095***	1071(27.71)	1933(50.01)	861(22.28)	***0.03746***
		rs6320			T	A		TT	TA	AA	
		SCZ	0.684	1.08	2759(64.83)	1497(35.17)	0.058870	890(41.82)	979(46.01)	259(12.17)	0.151378
		CTR	0.809	0.99–1.17	4877(63.09)	2853(36.91)		1542(39.9)	1793(46.39)	530(13.71)	

SCZ: schizophrenia; CTR: control; CI: confidence interval; OR: odds ratio.

^1^Risk allele and significant *P* values are in bold italics, and corrected *P* values are underlined after the Bonferroni correction (*P* × 30 in the discovery stage and *P* × 10 in the replication stage). A *P*-value of 0.05 was chosen as the threshold for significance.

^2^OR refers to the risk allele odds ratio in cases and controls.

**Table 3:** Common haplotype frequency and association analysis.

Haplotype	Haplotypic frequency in the discovery stage (%)				Haplotypic frequency in the replication stage (%)			
	SCZ	CTR	*P*-value^1^	Global *P*-value^2^	SCZ	CTR	*P*-value^1^	Global *P*-value^2^
*HTR1A* (rs878567-rs6449693- rs34118353-rs6294)								
***GAGC***	79.2	76.8	***0.042***	***<0.001***	80.2	78.2	***0.015***	***<0.001***
AGGC	8.56	10.5	***0.011***		8.93	9.96	0.069	
AGAT	7.26	7.62	0.512		7.83	8.24	0.344	
*HTR5A* (rs2873379-rs1017488-rs1881691-rs1800883)								
***TCAC***	46.5	51.6	***<0.001***	***<0.001***	48.5	52.1	***<0.001***	***<0.001***
CACG	35.0	36.4	0.265		36.7	38.0	0.176	
***TCAG***	10.4	6.81	***<0.001***		8.88	6.76	***<0.001***	
***TACG***	4.88	2.71	***<0.001***		3.94	1.50	***<0.001***	

SCZ: schizophrenia; CTR: control. Significant haplotypes and *P* values are in bold italics. Rare haplotypes are not shown if the frequency is less than 1%. A *P*-value of 0.05 was chosen as the threshold for significance.

^1^Based on 10000 permutations.

^2^Based on comparison of frequency distribution of all haplotypes for the combination of SNPs.

**Table 4:** Comparisons of executive function of 2 associated SNPs in patients and controls.

Subjects	SNP/Genotype	PE	NPE	TE	TCFC
Schizophrenia patients	rs878567 (*HTR1A*)				
	GG	31.52 (0.17)	38.73 (0.10)	70.24 (0.20)	67.57 (0.11)
	GA	31.95 (0.26)	38.50 (0.15)	70.44 (0.30)	67.55 (0.16)
	AA	32.85 (0.83)	39.33 (0.48)	72.18 (0.98)	67.60 (0.51)
	*P*-value	0.139	0.179	0.144	0.992
	rs1800883 (*HTR5A*)				
	CC	31.29 (0.28)	38.66 (0.16)	69.95 (0.33)	67.47 (0.18)
	CG	31.52 (0.20)	38.63 (0.12)	70.15 (0.24)	67.78 (0.12)
	GG	32.37 (0.27)	38.79 (0.15)	70.14 (0.32)	67.24 (0.17)
	*P*-value	***0.012***	0.725	***0.016***	***0.031***
	non-GG	31.44 (0.16)	38.64 (0.10)	70.08 (0.19)	67.68 (0.10)
	GG	32.37 (0.27)	38.79 (0.15)	70.14 (0.32)	67.24 (0.17)
	*P*-value	***0.004***	0.435	***0.005***	***0.028***
Healthy controls	rs878567 (*HTR1A*)				
	GG	5.630 (0.052)	13.689 (0.062)	19.319 (0.082)	22.171 (0.105)
	GA	5.470 (0.074)	13.713 (0.087)	19.184 (0.115)	21.898 (0.147)
	AA	5.822 (0.201)	13.625 (0.236)	19.447 (0.314)	22.268 (0.401)
	*P*-value	0.104	0.932	0.548	0.285
	rs1800883 (*HTR5A*)				
	CC	5.551 (0.08)	13.727 (0.09)	19.278 (0.12)	22.140 (0.16)
	CG	5.584 (0.06)	13.694 (0.07)	19.278 (0.093)	22.071 (0.12)
	GG	5.636 (0.09)	13.653 (0.10)	19.289 (0.14)	22.057 (0.18)
	*P*-value	0.77	0.867	0.998	0.923

PE: perseverative errors; NPE: non-perseverative errors; TE: total errors; TCFC: trials to compete first category. Data are shown as the mean, and all standard errors (SE) are indicated in the parentheses. *P* values were adjusted for age, gender, duration of illness and PANSS (Positive and Negative Syndrome Scale) in patients and for only age and gender in controls. A *P*-value of 0.05 was chosen as the threshold for significance, and significant *P* values are in bold italics.
