# Supplementary material for: Evaluation of association of common variants in HTR1A and HTR5A with schizophrenia and executive function
Source: Sci Rep. 2016 Nov 29;6:38048. doi: 10.1038/srep38048 (PMC5126681; doi:10.1038/srep38048)
Supplement: Supplementary Information [file srep38048-s1.pdf]

Title: Evaluation of association of common variants in *HTR1A* and *HTR5A* with schizophrenia and executive function

Author names and affiliations: Fanglin Guan [a,c], Huali Lin [b], Gang Chen [c], Lu Li [c], Teng Chen [a,c], Xinshe Liu [c,d], Jiuqiang Han[e] and Tao Li [c,d]

[a] Department of Forensic Psychiatry, School of Medicine & Forensics, Xi'an Jiaotong University, Xi'an, China;
[b] Xi’an Mental Health Center, Xi’an, China;
[c] Key Laboratory of National Ministry of Health for Forensic Sciences, School of Medicine & Forensics, Xi’an Jiaotong University, Xi’an, China;
[d] Department of Forensic Medicine, School of Medicine & Forensics, Xi’an Jiaotong University, Xi’an, China;
[e] School of Electronic and Information Engineering, Xi'an Jiaotong University, Xi'an, China.

Corresponding Author:

Tao Li, M.D. & Ph.D., Department of Forensic Medicine, School of Medicine & Forensics, Xi’an Jiaotong University, Xi’an, Shaanxi, China, 710061.

Tel: 86-29-82655117; Fax: 86-29-82655472. E-mail: litao050428@mail.xjtu.edu.cn

Table S1 Allele and genotype frequency of single SNP association analysis

Gene	Makers		Allele Freq. (%)		*P*-value	Genotype Freq. (%)			*P*-value	H-W E *P*-value
					The discovery stage					
HTR1A	rs1423691		C	T		CC	CT	TT		
		SCZ	1904(85.38)	326(14.62)	0.145621	816(73.18)	272(24.39)	27(2.42)	0.314089	0.447
		CTR	3968(86.68)	610(13.32)		1721(75.19)	526(22.98)	42(1.83)		0.806
	rs10052087		A	C		AA	AC	CC		
		SCZ	2021(90.63)	209(9.37)	0.226110	918(82.33)	185(16.59)	12(1.08)	0.351665	0.437
		CTR	4106(89.69)	472(10.31)		1840(80.38)	426(18.61)	23(1)		0.763
	rs6449693		A	G		AA	AG	GG		
		SCZ	1826(81.88)	404(18.12)	0.426517	749(67.17)	328(29.42)	38(3.41)	0.721550	0.777
		CTR	3712(81.08)	866(18.92)		1506(65.79)	700(30.58)	83(3.63)		0.882
	rs34118353		G	A		GG	GA	AA		
		SCZ	2032(91.12)	198(8.88)	0.516221	928(83.23)	176(15.78)	11(0.99)	0.643036	0.413
		CTR	4193(91.59)	385(8.41)		1920(83.88)	353(15.42)	16(0.7)		0.959
	rs6294		C	T		CC	CT	TT		
		SCZ	2025(90.81)	205(9.19)	0.200132	922(82.69)	181(16.23)	12(1.08)	0.420320	0.355
		CTR	4112(89.82)	466(10.18)		1850(80.82)	412(18)	27(1.18)		0.453
	rs75604552		C	T		CC	CT	TT		
		SCZ	2047(91.79)	183(8.21)	0.315981	942(84.48)	163(14.62)	10(0.9)	0.515801	0.322
		CTR	4234(92.49)	344(7.51)		1959(85.58)	316(13.81)	14(0.61)		0.746
HTR5A	rs1440454		C	T		CC	CT	TT		
		SCZ	1588(71.21)	642(28.79)	0.266234	563(50.49)	462(41.43)	90(8.07)	0.508720	0.724
		CTR	3319(72.5)	1259(27.5)		1204(52.6)	911(39.8)	174(7.6)		0.926
	rs9642644		G	A		GG	GA	AA		
		SCZ	1792(80.36)	438(19.64)	0.818301	723(64.84)	346(31.03)	46(4.13)	0.891798	0.571
		CTR	3668(80.12)	910(19.88)		1470(64.22)	728(31.8)	91(3.98)		0.942
	rs2919435		A	G		AA	AG	GG		
		SCZ	1865(83.63)	365(16.37)	0.543592	781(70.04)	303(27.17)	31(2.78)	0.813339	0.805
		CTR	3855(84.21)	723(15.79)		1623(70.9)	609(26.61)	57(2.49)		0.989
	rs2581842		G	A		GG	GA	AA		
		SCZ	1258(56.41)	972(43.59)	0.389270	358(32.11)	542(48.61)	215(19.28)	0.680277	0.700
		CTR	2532(55.31)	2046(44.69)		703(30.71)	1126(49.19)	460(20.1)		0.813
	rs2873379		T	C		TT	TC	CC		
		SCZ	1399(62.74)	831(37.26)	0.507167	442(39.64)	515(46.19)	158(14.17)	0.756892	0.685
		CTR	2834(61.9)	1744(38.1)		877(38.31)	1080(47.18)	332(14.5)		0.987
	rs1017488		C	A		CC	CA	AA		
		SCZ	1299(58.25)	931(41.75)	0.368743	374(33.54)	551(49.42)	190(17.04)	0.569920	0.593
		CTR	2719(59.39)	1859(40.61)		810(35.39)	1099(48.01)	380(16.6)		0.825
	rs1881691		A	C		AA	AC	CC		
		SCZ	1309(58.7)	921(41.3)	0.345644	380(34.08)	549(49.24)	186(16.68)	0.547184	0.605
		CTR	2742(59.9)	1836(40.1)		824(36)	1094(47.79)	371(16.21)		0.805
	rs2241859		G	T		GG	GT	TT		

		SCZ	1752(78.57)	478(21.43)	0.614409	691(61.97)	370(33.18)	54(4.84)	0.834342	0.622
		CTR	3621(79.1)	957(20.9)		1433(62.6)	755(32.98)	101(4.41)		0.902
	rs2581841		T	C		TT	TC	CC		
		SCZ	1679(75.29)	551(24.71)	0.097124	633(56.77)	413(37.04)	69(6.19)	0.253279	0.881
		CTR	3530(77.11)	1048(22.89)		1362(59.5)	806(35.21)	121(5.29)		0.901
	rs1561598		G	T		GG	GT	TT		
		SCZ	2111(94.66)	119(5.34)	0.189768	1001(89.78)	109(9.78)	5(0.45)	0.333507	0.279
		CTR	4367(95.39)	211(4.61)		2083(91)	201(8.78)	5(0.22)		0.948
	rs732050		A	G		AA	AG	GG		
		SCZ	1726(77.4)	504(22.6)	0.518799	670(60.09)	386(34.62)	59(5.29)	0.742446	0.726
		CTR	3575(78.09)	1003(21.91)		1394(60.9)	787(34.38)	108(4.72)		0.819
	rs2698512		G	A		GG	GA	AA		
		SCZ	1880(84.3)	350(15.7)	0.249314	794(71.21)	292(26.19)	29(2.6)	0.486936	0.729
		CTR	3809(83.2)	769(16.8)		1584(69.2)	641(28)	64(2.8)		0.930
	rs1657268		T	C		TT	TC	CC		
		SCZ	1392(62.42)	838(37.58)	0.680043	436(39.1)	520(46.64)	159(14.26)	0.916445	0.844
		CTR	2834(61.9)	1744(38.1)		879(38.4)	1076(47.01)	334(14.59)		0.873
	rs1730208		G	A		GG	GA	AA		
		SCZ	1707(76.55)	523(23.45)	0.243534	656(58.83)	395(35.43)	64(5.74)	0.463836	0.656
		CTR	3562(77.81)	1016(22.19)		1385(60.51)	792(34.6)	112(4.89)		0.929
	rs1946915		T	C		TT	TC	CC		
		SCZ	1864(83.59)	366(16.41)	0.131405	776(69.6)	312(27.98)	27(2.42)	0.279181	0.508
		CTR	3759(82.11)	819(17.89)		1543(67.41)	673(29.4)	73(3.19)		0.971
	rs1631327		C	T		CC	CT	TT		
		SCZ	1791(80.31)	439(19.69)	0.438783	716(64.22)	359(32.2)	40(3.59)	0.655734	0.543
		CTR	3640(79.51)	938(20.49)		1447(63.22)	746(32.59)	96(4.19)		0.990
	rs2581831		C	T		CC	CT	TT		
		SCZ	1796(80.54)	434(19.46)	0.101520	725(65.02)	346(31.03)	44(3.95)	0.259640	0.736
		CTR	3762(82.18)	816(17.82)		1547(67.58)	668(29.18)	74(3.23)		0.855
	rs1371818		T	C		TT	TC	CC		
		SCZ	1812(81.26)	418(18.74)	0.095578	733(65.74)	346(31.03)	36(3.23)	0.197154	0.532
		CTR	3795(82.9)	783(17.1)		1575(68.81)	645(28.18)	69(3.01)		0.764
	rs2581832		C	A		CC	CA	AA		
		SCZ	1824(81.79)	406(18.21)	0.528699	749(67.17)	326(29.24)	40(3.59)	0.782924	0.541
		CTR	3773(82.42)	805(17.58)		1557(68.02)	659(28.79)	73(3.19)		0.748
	rs1730215		A	G		AA	AG	GG		
		SCZ	1389(62.29)	841(37.71)	0.491913	437(39.19)	515(46.19)	163(14.62)	0.661591	0.573
		CTR	2812(61.42)	1766(38.58)		861(37.61)	1090(47.62)	338(14.77)		0.817
					The replication stage					
HTR1A	rs6449693		A	G		AA	AG	GG		
		SCZ	3469(81.51)	787(18.49)	0.681539	1411(66.31)	647(30.4)	70(3.29)	0.822912	0.691
		CTR	6277(81.2)	1453(18.8)		2551(66)	1175(30.4)	139(3.6)		0.797
	rs34118353		G	A		GG	GA	AA		
		SCZ	3869(90.91)	387(9.09)	0.475538	1762(82.8)	345(16.21)	21(0.99)	0.691620	0.372

		CTR	7057(91.29)	673(8.71)		3223(83.39)	611(15.81)	31(0.8)		0.730
	rs6294		C	T		CC	CT	TT		
		SCZ	3852(90.51)	404(9.49)	0.489667	1747(82.1)	358(16.82)	23(1.08)	0.398306	0.334
		CTR	6966(90.12)	764(9.88)		3135(81.11)	696(18.01)	34(0.88)		0.498
HTR5A	rs2873379		T	C		TT	TC	CC		
		SCZ	2648(62.22)	1608(37.78)	0.376385	826(38.82)	996(46.8)	306(14.38)	0.667305	0.837
		CTR	4746(61.4)	2984(38.6)		1457(37.7)	1832(47.4)	576(14.9)		0.998
	rs1017488		C	A		CC	CA	AA		
		SCZ	2499(58.72)	1757(41.28)	0.142597	726(34.12)	1047(49.2)	355(16.68)	0.270212	0.493
		CTR	4645(60.09)	3085(39.91)		1399(36.2)	1847(47.79)	619(16.02)		0.820
	rs1881691		A	C		AA	AC	CC		
		SCZ	2477(58.2)	1779(41.8)	0.246573	715(33.6)	1047(49.2)	366(17.2)	0.419949	0.605
		CTR	4583(59.29)	3147(40.71)		1364(35.29)	1855(47.99)	646(16.71)		0.719

SCZ: schizophrenia; CTR: control

Table S2 Summary of the association test for 7 imputed and typed SNPs with nominal significance

Source	SNP ID	Chromosome	Position	Allele A	Allele B	MAF	*P*-value	Gene
Imputed	rs986128	5	62145263	C	T	0.090723	0.020999	185kb from *IPO11*
Typed	rs878567	5	63291747	A	G	0.195358	0.001538	*HTR1A*
Typed	rs6295	5	63294321	C	G	0.250441	0.018729	*HTR1A*
Imputed	rs10940011	5	64210104	C	T	0.130445	0.024253	*CWC27*
Imputed	rs1309580	5	64309150	A	G	0.127804	0.032462	*CWC27*
Typed	rs1800883	7	154493524	C	G	0.482667	0.000251	*HTR5A*
Typed	rs6320	7	154493554	A	T	0.36839	0.017278	*HTR5A*

MAF: minor allele frequency

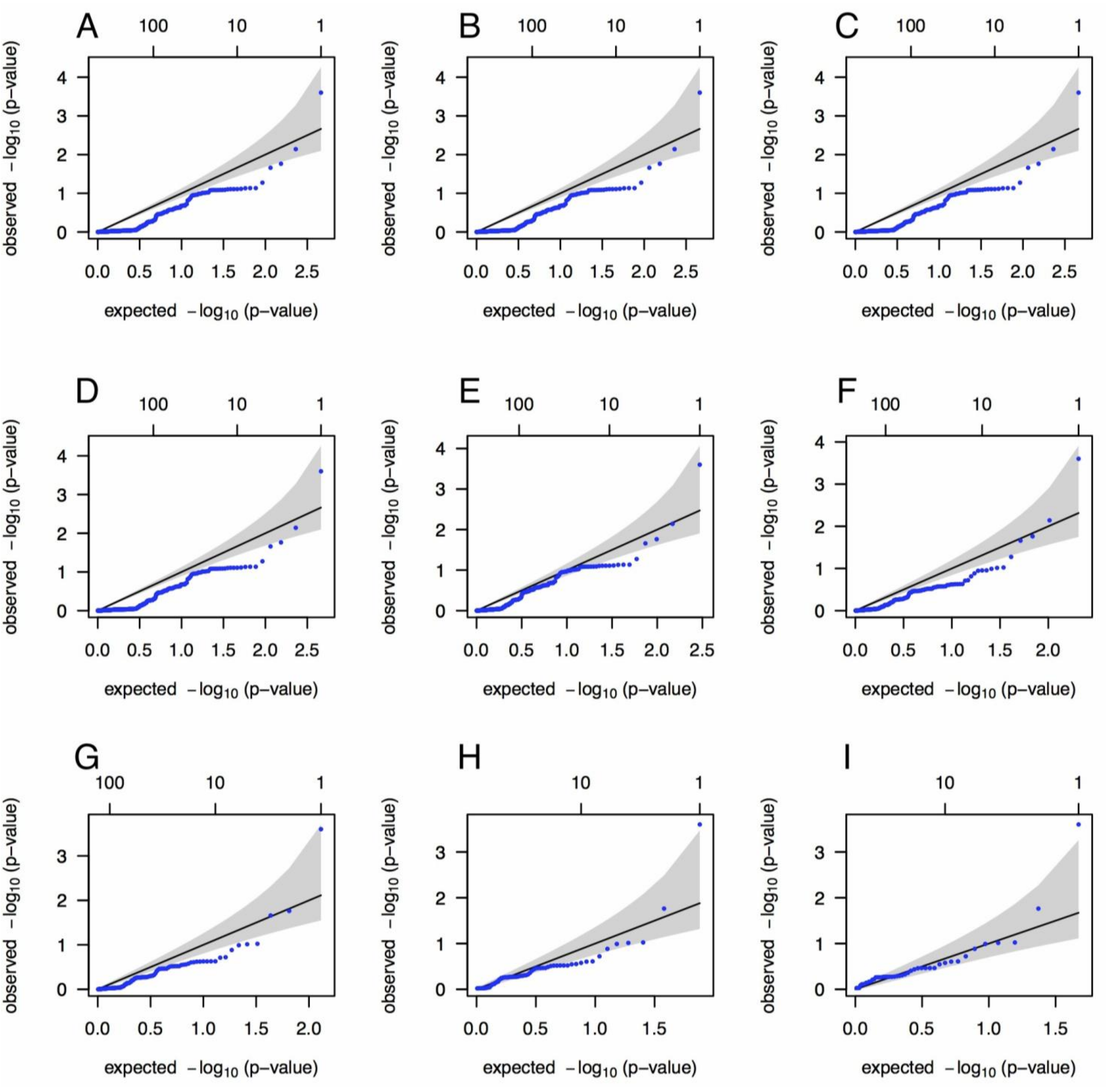

Figure S1. The Q-Q plots made by association test results based on marker set filter by different certainty thresholds. The certainty thresholds used in A, B, C, D, E, F, G, H, I are 0.1, 0.2, 0.3, 0.4, 0.5, 0.6, 0.7, 0.8, 0.9 respectively. No significant deviance from expected line could be found when certainty threshold was chosen as 0.8.
